# Biodegradable Polyvinyl Alcohol (PVOH)-Based Films with Anthocyanin-Rich Extracts of Corozo (*Bactris guineensis* H.E. Moore) for Intelligent Packaging Design

**DOI:** 10.3390/polym17070933

**Published:** 2025-03-29

**Authors:** Fabián Rico-Rodríguez, Alexis López-Padilla, Rodrigo Ortega-Toro

**Affiliations:** 1 Grupo de Investigación en Transformación Aplicada a Matrices Industriales y Agroindustriales—ITMIA, Food Engineering Department, Faculty of Engineering, Universidad de Cartagena, Cartagena de Indias 130015, Colombia; fricor@unicartagena.edu.co (F.R.-R.); alopezp2@unicartagena.edu.co (A.L.-P.); 2 Food Packaging and Shelf-Life Research Group (FP&SL), Food Engineering Department, Universidad de Cartagena, Cartagena de Indias 130015, Colombia

**Keywords:** anthocyanin extraction, biodegradable film, Corozo extract, structural properties, polyvinyl alcohol

## Abstract

Corozo (*Bactris guineensis* H.E. Moore) is a fruit from the Colombian Caribbean region valued for its thermostable anthocyanins, which are responsible for its characteristic reddish colour. This study aimed to evaluate the physicochemical, structural, and functional properties for an intelligent and biodegradable film design based on a polyvinyl alcohol (PVOH) matrix incorporating a Corozo extract rich in anthocyanins, with potential applications in food packaging. Anthocyanins were extracted from Corozo fruit and evaluated throughout a central composite design (CCD) for the effects of three variables—extraction time (t), temperature (T), and solvent concentration (CS). A quadratic model (R^2^ = 0.9586) demonstrated that the exocarp (peel) was the most effective source of anthocyanins. The best conditions were a 1:16.66 solid-to-solvent ratio at 50 °C for 75 min, yielding 38.65 mg EC_3_G/L. PVOH films were formulated using Corozo anthocyanin extract (CAE), which was characterised for the total anthocyanin content. Characterisation of the films revealed that the incorporation of Corozo-derived phenolic compounds did not cause significant (*p* < 0.05) changes in structural or water interaction properties compared to those of the control sample.

## 1. Introduction

Corozo, also known as ’Uva de lata’ (*Bactris guineensis* H.E. Moore), is a palm fruit native to the Colombian Caribbean region and Central America valued for its nutritional and sensory properties. Each corozo palm produces approximately 30 kg of fruit per year, contributing to an estimated yield of 750 kg per hectare [1]. This species thrives in dry areas below 100 m above sea level. The reddish-black, semi-spherical fruits grow in clusters of 50 to 100 units. In Colombia, corozo is primarily processed into juices, fermented beverages, jams, and desserts, with the husk and seed discarded as by-products [2,3].

Beyond its nutritional and sensory appeal, corozo is recognised for its high anthocyanin content, which makes it a potential source of natural pigments. Its characteristic reddish colour is attributed to thermostable anthocyanins, primarily cyanidin-3-glucoside (72.2%) and cyanidin-3-rutinoside (15.7%) [4,5]. These natural pigments exhibit high stability, making them suitable for applications in food packaging, pharmaceuticals, and the textile industry. Anthocyanins, a subclass of phenolic compounds, possess high antioxidant activity. Notably, cyanidin-3-glucoside has demonstrated potent antioxidant properties in various biological systems, with the ability to neutralise free radicals, thereby mitigating oxidative stress and its associated cellular damage [6].

However, corozo fruit processing generates substantial by-products, including husks, seeds, and residual pulp, which remain rich in anthocyanins. These anthocyanins not only function as antioxidants but also exhibit halochromic behaviour, meaning that their colour changes in response to environmental pH. This property makes them valuable for intelligent packaging applications [7]. The ability of anthocyanins to alter their colour in response to external stimuli, such as light, temperature, or pH, has been harnessed in the development of chromatic materials. Intelligent packaging leverages this phenomenon, allowing phenolic compounds to serve as pH-sensitive indicators.

Similarly, commercial pH indicators, which rely on halochromic dyes embedded in polymer-based matrices, share similarities with laboratory-level innovations. These indicators typically consist of polymer films fabricated using methods such as casting or compression moulding [8]. However, environmental concerns regarding petroleum-based plastics have heightened interest in biodegradable alternatives.

Polyvinyl alcohol (PVOH) is a promising biodegradable polymer due to its water solubility, high biodegradability, and ability to form film solutions. PVOH-based films are already utilised in the food industry for the preservation of refrigerated meats [9].

The properties of PVOH, including tensile strength and water resistance, depend on its molecular weight and degree of hydrolysis. Higher molecular weight and hydrolysis levels enhance properties such as tensile strength, water resistance, barrier effectiveness, and solvent resistance while reducing flexibility, solubility, and water sensitivity [10,11]. Despite the promising properties of anthocyanins and their potential applications in biodegradable packaging, research on the incorporation of corozo-derived anthocyanins into PVOH-based intelligent films remains limited. This study aimed to optimise extraction conditions and evaluate the physicochemical, structural, and functional properties of biodegradable films formulated with corozo anthocyanin extract. These films offer an eco-friendly alternative to conventional plastics while preserving the bioactive properties of anthocyanins. Furthermore, the study highlights the potential of *Bactris guineensis* as an underexplored natural resource for the development of pH-sensitive, anthocyanin-rich films in sustainable food packaging applications.

## 2. Materials and Methods

### 2.1. Reagents

Corozo (*Bactris guineensis* H.E. Moore) was obtained from a local market in Cartagena de Indias, Colombia. The following two fractions were obtained from corozo: the peel (F_C_) and the pulp (F_P_). The fractions were dried at 55 °C until a constant mass was achieved. The peel was ground, and both fractions were stored at 4 °C in darkness until further use. The reagents and equipment for the extraction process (absolute ethanol and citric acid) were provided by the University of Cartagena.

### 2.2. Extraction of Anthocyanins

The extractions were carried out using a solvent (acidified ethanol) by mixing 2 g of citric acid with 50 mL of water and adding 48 mL of ethanol [12]. A central composite design (CCD) was employed to determine the best extraction conditions based on three factors affecting total anthocyanin content (TAC)—time (t), temperature (T), and the solvent-to-solute ratio (AS). See Table 1. The extractions were conducted in darkness using a constant volume of solvent and heating plates (IKA C-MAG HS7; IKA Werke GmbH & Co. KG, Staufen im Breisgau, Germany). Following extraction, vacuum filtration was carried out using ASTM E11 [13] with #80 and #200 meshes, manufactured by Advantech Manufacturing Inc., Deerfield, IL, USA. The samples were then centrifuged for 10 min at 1967.68× *g* (Artilab Z 206 A centrifuge HERMLE Labortechnik GmbH, Wehingen, Germany), and the supernatant was collected and stored in 50 mL Falcon tubes at −18 °C for 24 h to precipitate the soluble solids present in the suspension. Afterward, the supernatant was extracted using a 5 mL micropipette, and the extract was stored in 500 mL amber containers at −4 °C until further use to protect it from light degradation.

### 2.3. Characterisation of the Extract

#### 2.3.1. Halochromic Response of the Extracts

The halochromic responses of the F_C_ and F_P_ extracts were analysed using McIlvaine buffer solutions [14] prepared by mixing 0.2 M disodium hydrogen phosphate with 0.1 M citric acid. The pH scale ranged from 3.0 to 8.0, and a 1:5 solvent-to-solute ratio was used to visualise the colour change.

#### 2.3.2. Determination of the Total Anthocyanin Content (TAC)

The TAC was determined using the pH-differential method. Two samples from each extract (F_C_ and F_P_) were taken and mixed with buffer solutions at pH = 1.0 (KCl 0.025M/HCl) and another at pH = 4.5 (CH_3_CO_2_Na 0.4M/HCl), both adjusted to the required pH with 0.1M HCl. Absorbance measurements were performed in duplicate (*n* = 2) at a wavelength of 520 nm and then at 700 nm to eliminate extract turbidity using a spectrophotometer BIOBASE BK-UV1900 (BIOBASE, Jinan, China) with 3.5 mL glass cuvettes. The *TAC* was subsequently calculated using Equation (1) [15].

(1) $$TAC=\frac{A\times MW\times DF}{\left(\varepsilon \times l\right)}\times 1000$$

where, A = (A_520_ − A_700_) pH 1.0–(A_520_ − A_700_) pH 4.5. MW is the molecular weight (449.2 g/mol). DF is the dilution factor, ε is the molar absorptivity (26.900), and *l* is the path length (1 cm). The DF was obtained by diluting the samples until the absorbance value at 520 nm was within the ideal range (0.2 to 1.4 AU).

#### 2.3.3. FTIR Analysis of Corozo Anthocyanin Extract

The FTIR analysis was performed using an infrared spectrophotometer Buck Scientific infrared detector (Buck Scientific Instruments, LLC, Ansonia, CT, USA) for M530 with a scan range from 600 cm^−1^ to 4000 cm^−1^. To ensure the accuracy and reliability of the measurements, air was used as the background for the blank measurement, eliminating interference from moisture and carbon dioxide present in the environment.

#### 2.3.4. Film Preparation

The film-forming solution (FFS) was prepared following the formulations defined in Table 2. Three formulations of PVOH-based films with corozo anthocyanin extract (CAE) were prepared by casting; additionally, a control film was prepared without extract (pure PVOH), which was prepared by dissolving 4 g of PVOH in 200 mL of distilled water and homogenised using magnetic stirring. Glycerol was added as a plasticizer at 20% (*w*/*w*) relative to the polymer, and the mixture was stirred for 15 min. The solution was then degassed under a vacuum, filtered, poured into Teflon trays, and dried at 40–45 °C for 9 h. This process was repeated for formulations F1, F2, and F3, replacing part of the water in the solution with CAE according to the formulation (10%, 20%, and 30%). The films were stored in desiccators with a saturated sodium bromide solution at a relative humidity (RH) of 53% and 30 °C to condition for a period of 3–5 days.

#### 2.3.5. Optical Properties

The colour of the films was measured with a portable colourimeter Colourimeter (CHN Spec CS-10, CHN Spec Technology Co., Ltd., Hangzhou, China). The device provided CIE Lab* coordinates, along with hue angle (h) and chroma (c), also known as saturation. In the CIE Lab* system, lightness (L*) is represented on the vertical axis, while the horizontal axes indicate chromatic orientation towards red, green, blue, or yellow [16].

To assess colour variation, a reference sample was used. The colour difference for each coordinate (L*, a*, and b*) was calculated, and the overall colour difference (ΔE*) was determined using Equation (2):

(2) $$∆E^{*}=\sqrt{{{∆L}^{*}}^{2}+{{∆b}^{*}}^{2}+{{∆a}^{*}}^{2}}$$

Gloss was measured at a 60° angle in accordance with ASTM D523 [17,18] using a 3NH YG268 (Minolta, Langenhagen, Germany) multi-angle gloss meter. A total of six films were analysed, each measured in triplicate (n = 3). Results were expressed in gloss units (GU).

#### 2.3.6. Moisture Content (MC), Water Absorption Capacity (WAC), and Contact Angle (CA)

The methodology proposed by Rezaei et al. [19] was followed to determine the moisture content and water solubility of the films. Film samples (2 cm × 2 cm) were first weighed (W0) and then dried in an oven at 60 °C until a constant weight (W1) was reached. After drying, the samples were immersed in distilled water at a 1:10 ratio (film/water) for 24 h. Subsequently, the films were removed dried again to a constant weight (W2), and Equation (3) was applied to calculate moisture content.

(3) $$\%\;Moisture=\frac{W1-W2}{W1}\times 100\%$$

Water absorption capacity was determined according to the protocol outlined by Pirsa [20]. Film samples (2 cm × 2 cm) were placed in a desiccator containing calcium chloride to maintain 0% relative humidity at room temperature. The samples were weighed at 24-h intervals until a stable weight was reached, representing the dry film mass (W_s_). After this, the samples were transferred to a desiccator containing a saturated potassium sulphate solution and weighed were weighed every 24 h until equilibrium was reached. The final mass was recorded as the wet film mass (W_h_), and water absorption capacity was calculated using Equation (4):

(4) $$\%\;Water\;absorption\;capacity=\frac{W_{h}-W_{s}}{W_{s}}\times 100\%$$

For contact angle measurements, a 2 × 2 cm film sample was placed on a horizontal surface with a white background, and a dyed water droplet was deposited on its surface. The image was captured with a digital camera after 30 s, maintaining a fixed 20 cm distance between the droplet and the camera lens. The image was analysed using Goniotrans Software (version 1.0.3). This procedure was conducted in triplicate for each formulation, with the mean and standard deviation reported [21].

Water vapour permeability (WVP) was determined using a gravimetric approach following the methodology of Aguirre et al. (2013) [22], with modifications [19]. Environmental conditions were adjusted to establish a humidity gradient between 52.8% and 100% relative humidity at a controlled temperature of 25 °C. Only films without physical imperfections were used for WVP evaluations. Payne-type permeation vessels filled with distilled water were used, exposing one film surface to 100% relative humidity. These vessels were placed in humidity-controlled cabinets at 25 °C, with relative humidity maintained at 52.8% using supersaturated magnesium nitrate solutions.

To improve the applicability of the films for high-water-activity products, the free film surface was exposed to a lower relative humidity environment during manufacture. The vessels containing the films were systematically weighed using a high-precision analytical balance (sensitivity: 0.0001 g). Once a stable measurement condition was reached, the water vapour transmission rate (WVTR) was calculated from the slope of the regression line describing weight loss over time. This value was then normalised by the film surface area. The procedure was repeated three times, and results were reported as the mean value and standard deviation.

#### 2.3.7. Structural Properties

Fourier-transform infrared (FTIR) analysis was conducted following the methodology described by Madadi et al. [23] Infrared spectroscopy measurements were performed using an FTIR spectrophotometer over a frequency range of 500 cm^−1^ to 4000 cm^−1^.

Internal transmittance was determined using the full UV-Vis spectrum using film samples (1 cm × 3 cm) conditioned at 75% relative humidity (RH) at 25 °C. A UV-Vis spectrophotometer (BIOBASE BK-UV1900, BIOBASE Group, Jinan, China) was used to record transmittance over a wavelength range of 200 nm to 1000 nm [20].

Film opacity was measured at 600 nm using a UV-Vis spectrophotometer (BIOBASE BK-UV1900), with triplicate measurements (n = 3) taken for each sample. Film dimensions were 0.8 mm × 40 mm. Opacity was calculated using Equation (5) [17].

(5) $$Opacity=\frac{{Abs}_{600}}{x}$$

where, Abs_600_ = Absorbance value at 600 nm and x = film thickness in mm.

Film thickness was determined based on eight random measurements per sample. The average and standard deviation were then calculated. Each film was analysed in triplicate (n = 3) to ensure data reliability.

Microstructural analysis of the film cross-sections and surfaces was performed using a scanning electron microscope (JSM-5910, JEOL Ltd., Tokyo, Japan). Film samples were maintained in desiccators with P_2_O_5_ for two weeks to eliminate moisture. Film pieces (0.5 cm^2^) were cryofractured, mounted on copper stubs, gold-coated, and observed under an accelerating voltage of 10 kV.

#### 2.3.8. Statistical Analysis

Statistical analysis for the extraction process was conducted using DesignExpert^®^ software (version 23.1.8). Data obtained from film characterisation were subjected to analysis of variance (ANOVA) using a completely randomised factorial design. Multiple comparisons were performed using Tukey’s test, with significance set at *p* < 0.05.

## 3. Results

### 3.1. Effect of Concentration on the Halochromic Response of the CAE

**Figure 1:**
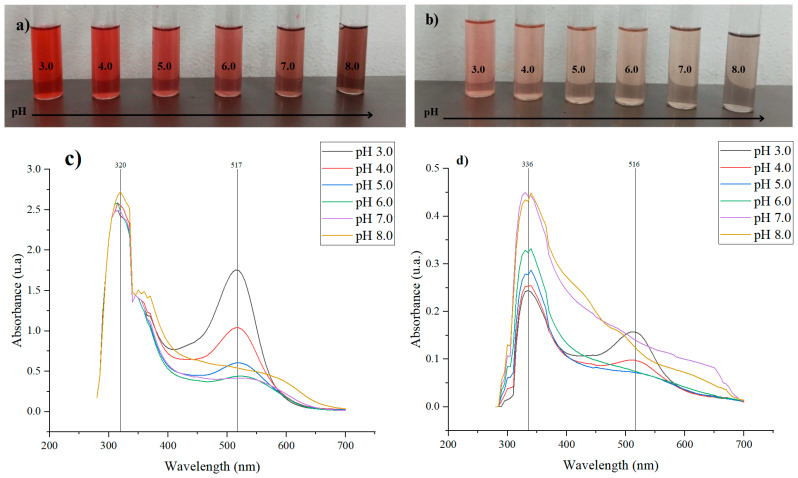
Colour variation of the (**a**) pulp extract (F_P_) and (**b**) peel extract (F_C_) diluted in McIlvaine buffer solutions (1:5) at pH 3.0–8.0. UV-Vis spectrum of the (**c**) pulp extract (F_P_) and (**d**) peel extract (F_C_) from 250 nm to 700 nm.

The ability of the extracts to function as pH indicators was initially evaluated by varying their concentration to assess the intensity of colour change. This is because the colour of anthocyanins is influenced by their concentration, as well as their dependence on pH. At lower concentrations, the colour change is more pronounced and occurs more rapidly [24,25]. The observed colour variations result from structural changes in the anthocyanin molecules in response to pH. The λmax readings indicate a shift, as the chromophore absorbs light at different wavelengths corresponding to the colour changes (see Figure 1).

The halochromic behaviour of fractions F_C_ and F_P_ is directly related to the ionic structure of anthocyanins, whose colour varies with the pH of the solution. Under acidic conditions, anthocyanins exhibit a red tendency due to the formation of flavylium cations, which are stable and highly soluble in water in such environments [26]. This was observed in fractions F_C_ and F_P_, where at pH 3.0–4.0, they appear reddish and pinkish, respectively. As the pH increases, anthocyanins undergo a structural transformation, shifting to a neutral purple state, and in more alkaline conditions, they form yellow chalcone structures through the opening of the C-ring [26,27]. In this context, F_C_ exhibited a transition toward brown hues, while F_P_ displayed a cream colour at neutral pH and grayish–brown shade at pH 8.0, reflecting the influence of pH on the molecular structure of anthocyanins, as shown in Figure 1a,b.

### 3.2. Total Anthocyanin Content

The effects of the different extraction conditions on anthocyanins yield from corozo pulp (F_P_) and peel (F_C_) are presented in Table 3. The total anthocyanin content (TAC) was quantified using the pH-differential method, which is widely used for phenolic compound analysis. This method relies on the quantification of cyanidin-3-O-glucoside, the predominant flavonoid in the sample [4,28,29].

A trend was observed where the TAC increased under conditions with a higher solute-to-solvent ratio (*m*/*v*). This is evident in Table 3, where treatments C5 and C15 exhibited the highest TAC values, suggesting that a greater solute ratio facilitates anthocyanin extraction. Conversely, treatment C10 showed a significantly lower TAC, aligning with previous predictions. A study by Vidana Gamage and Choo [29] using 1:10, 1:15, and 1:20 solid-liquid ratios found that the 1:15 ratio yielded the highest TAC. Similarly, Ku and Mun [30] reported TAC values of 37.2 at a solid-liquid ratio of 20, whereas a ratio of 10 yielded the lowest value of 27.9.

The choice of solvent also played a crucial role. As Chandrasekhar et al. [31] reported, anthocyanins exhibit greater stability at lower pH levels. To enhance extraction stability, citric acid was used to acidify the solvent. Moreover, statistical analysis identified treatment D as the best condition, with a 77.4% desirability score. The observed TAC closely matched predicted values, reinforcing its potential for efficient anthocyanin extraction.

A study by Erşan et al. [32] reported anthocyanin concentrations in corozo (*Bactris guineensis* (L.) H.E. Moore) of 4.8 ± 2.3, 132 ± 52, and 12 ± 3 and 0. 05 ± 0.04, 1.6 ± 1.2, and 0.21 ± 0.14 for peel (exocarp) and pulp (mesocarp) cyanidin-3-O-glucoside, cyanidin-3-O-ruthinoside and peonidin-3-O-ruthinoside, respectively. The best treatment with respect to extraction conditions was calculated by statistical analysis with DesingExpert software (version 23.18), where desirable conditions that maximised TAC and decreased extraction time were specified, giving a desirability of 77.4% where the conditions were (1:10, 30 min, 60 °C), yielding a predicted TAC of 17.89, which closely matched the experimental value 17.45 for the F_C_ fraction the F_P_ fraction was excluded from further analysis due to high sugar content, which compromised the reliability of TAC calculations.

### 3.3. Response Surface Methodology (RSM) and Face-Centred Cube Design (FCD)

**Figure 2:**
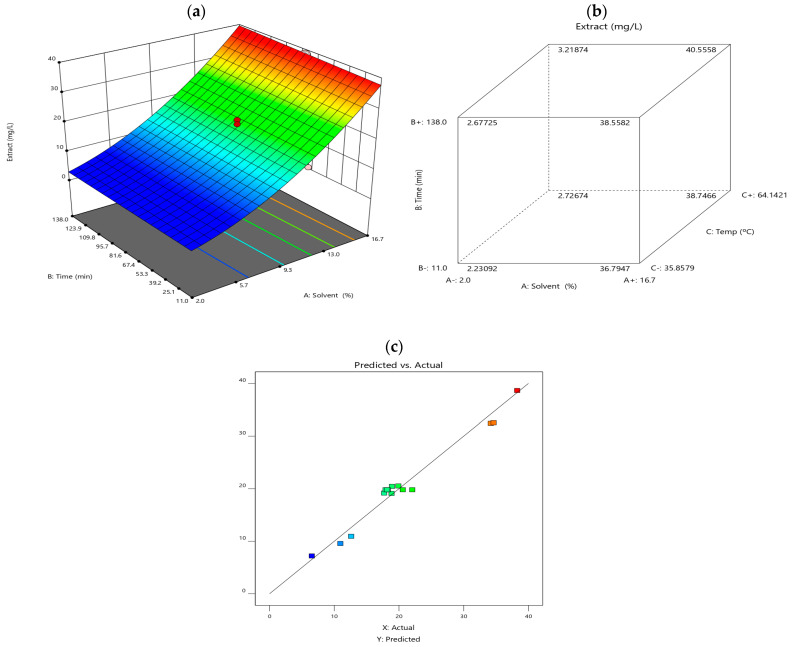
(**a**) illustrates the response surface plot, (**b**) cube plot of the combined effect of extraction variables (solvent, time, and temperature) on the anthocyanin content obtained, and (**c**) predicted vs. actual values.

Response surface methodology (RSM) was employed to evaluate the interactions between key extraction factors like the temperature, solid-liquid ratio and extraction time, to optimise anthocyanin yield. The results are visually represented in Figure 2. Diagram (a) illustrates the RSM model, showing the effect of different extraction variables on anthocyanin content. Diagram (b) depicts a face-centred cube design (FCD), highlighting interactions between solvent type, extraction time, and temperature. Diagram (c) compares predicted vs. experimental values, demonstrating the accuracy of the statistical model.

### 3.4. FTIR Analysis of Corozo Anthocyanin Extract

**Figure 3:**
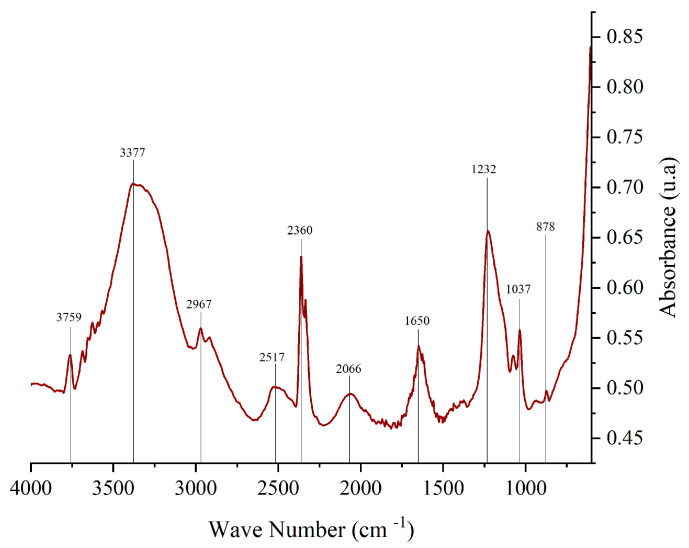
FTIR spectrum of anthocyanin extract from corozo hulls.

Fourier-transform infrared spectroscopy (FTIR) is widely used to identify functional groups in various compounds. Figure 3 presents the FTIR spectrum of corozo peel anthocyanin extract, highlighting key absorption peaks, as follows: 878.45 cm^−1^ corresponds to aromatic C-H bonds in the A and B rings of the anthocyanin structure; 1650.21 cm^−1^ is associated with C=C, C=N, and C=O bonds in the benzene ring; 2800 cm^−1^–3600 cm^−1^: functional groups present include C-H, O-H, N-H, C=C, C-O-C, and C=O, characteristic of anthocyanins; 1000 cm^−1^–1750 cm^−1^: ionic forms of anthocyanins are detected within this range; and 1231.89 cm^−1^: attributed to pyran rings, a key structural feature of flavonoids. The values found in the range between 1600 cm^−1^ and 1700 cm^−1^ suggests the presence of quinoidal forms of the anthocyanin molecule. 2841 cm^−1^–2943 cm^−1^ corresponds to C-H stretching, indicative of sugar groups in anthocyanins. The recorded value (2967 cm^−1^) is consistent with literature findings [33,34].

The observed spectra for anthocyanins extracted from corozo exhibited correlation with literature sources [35,36].

### 3.5. Characterisation of PVOH Films with CAE

#### 3.5.1. Optical Properties

The surface analysis results of polyvinyl alcohol (PVOH) films incorporating corozo anthocyanin extract (CAE) are presented in Table 4. Using the CIELab L* (light-dark), a* (red-green) and b* (yellow-blue) scales, consistent values were observed with respect to the visual colouration of the films. The control PVOH films exhibited an L* value of 92.25 ± 0.16, indicating a high degree of lightness. As the CAE content increased, the L* values decreased, reflecting a darker film appearance. Simultaneously, the a* parameter shifted toward positive values, confirming the strong reddish colouration induced by CAE, with values ranging from 21.21 to 55.36.

Additionally, the total colour difference (ΔE) increased proportionally with CAE concentration compared to the control sample (C), with values ranging from 25.59 to 66.21. This trend confirms that CAE incorporation significantly influences film colouration, reinforcing its potential application in pH-responsive and biodegradable packaging materials.

The gloss values of the films were measured at an incidence angle of 60°, which correlates with surface morphology and drying conditions. A smoother surface results in a higher gloss level [37]. The films exhibited low gloss values (ranging from 9 to 15 gloss units, GU), likely due to surface imperfections. According to Acevedo-Puello et al. [38] gloss can be influenced by roughness, surface uniformity, imperfections, and lighting conditions.

A relationship between optical properties and pH was also observed, highlighting the halochromic behaviour of anthocyanins. At low pH (acidic, F1 formulation): The films displayed more intense colours, reduced gloss (9.13 GU), and a high ΔE (25.59), with a reddish hue. At near-neutral pH (F2 formulation): The films appeared lighter, with increased gloss (15.26 GU) and ΔE (44.54), transitioning to purple shades with improved colour stability. At higher pH (alkaline, F3 formulation): The films shifted toward yellow shades, had a slightly reduced gloss (14.66 GU), and exhibited the highest ΔE (66.21), indicating structural changes in anthocyanins at alkaline pH [39,40]. These findings confirm that pH significantly influences the colour intensity and gloss of the films, further reinforcing the role of CAE as a natural pH-responsive indicator.

#### 3.5.2. Moisture Content (MC), Water Absorption Capacity (WAC) and Contact Angle (CA)

The moisture content (MC) of the films, conditioned at 53% relative humidity (RH), is presented in Table 5. The highest MC value was observed in the control PVOH film (23.98%). The addition of CAE (10%, 20%, 30%) did not significantly alter the MC, suggesting that CAE does not impact the water retention capacity of the films. These results align with those reported by Forghani et al. [41] (14.54% to 17.68%) and Yao et al. [42] (21.20% to 26.94%) for PVOH films enriched with betacyanins.

The contact angle (CA) values confirmed the hydrophilic nature of the PVOH films. Regardless of CAE concentration, the films showed no significant improvement in water repellency, with CA values ranging between 26.33° and 24.66° (Table 5). This range is consistent with results reported by Rahmadiawan et al. [43].

The water contact angle is a good indicator of the degree of hydrophilicity of the films, the behaviour of a water droplet demonstrates the surface wettability and surface energy. The CA values determine the hydrophobicity or hydrophilicity of polymeric matrices and various materials, depending on the solvents used and whether they are polar or apolar, greatly influencing the specific behaviour of water, a CA below 90° indicates a hydrophilic surface, influenced by the high density of hydroxyl (-OH) groups in PVOH and CAE, which promotes water affinity. A CA above 90° typically suggests a hydrophobic surface, while values exceeding 120° indicate superhydrophobicity [44,45].

Moisture is an extremely important factor in food storage which must be controlled since moisture content either in the packaging or in the environment can damage the food matrix, because it promotes microbial growth due to high water activity (aw) due to increased humidity, thus, the shelf life of the product is significantly reduced [20,43].

**Figure 4:**
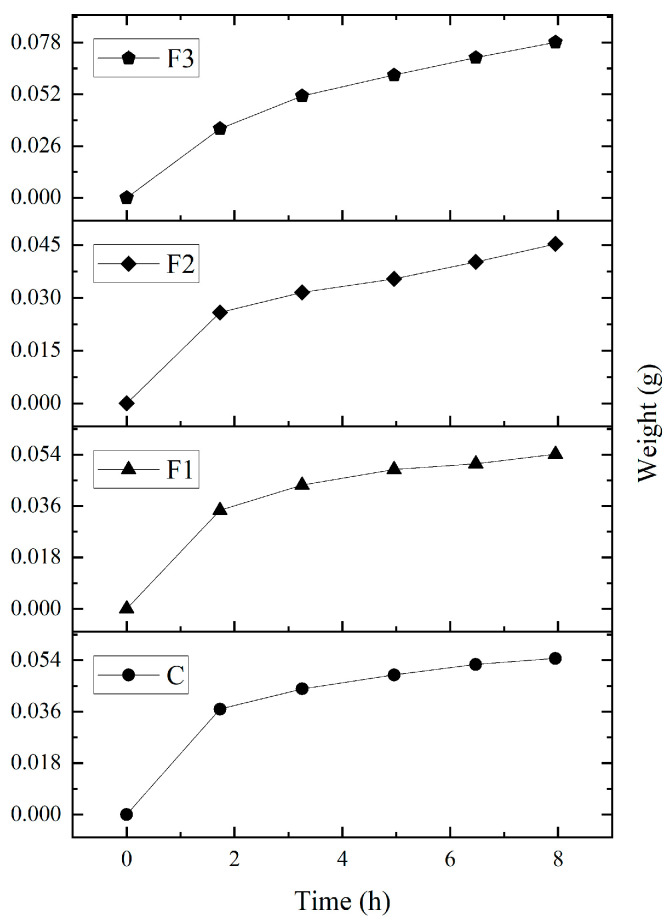
Moisture absorption capacity of PVOH and CAE films over a period of approximately 8 h, under 100% RH conditions.

The water absorption capacity (WAC) results (see Table 6) revealed no significant differences between formulations. The films absorbed 0.32–0.39 g of water per gram of dry film, corresponding to an average WAC of 34.5% over 7 h 30 min at 100% RH (Figure 4). Studies by Rahmadiawan et al. [43] found lower moisture absorption values due to thermal treatments which alter hydroxyl groups in the polymeric matrix and reduce hydrophilicity when were exposed to a RH of 75%.

The water vapor permeability (WVP) data (Table 6) showed no significant changes with CAE addition. PVOH films inherently lack strong water vapor barrier properties, as previously reported by Xu et al. [46] that showed WVP values of 0.71, this value is slightly higher than that reported in this research but suggests the same hydrophilic behaviour and reduced effectiveness as a water vapor barrier of PVOH. Several studies emphasise that the addition of anthocyanins to polymeric materials increases permeability due to the polar nature of these phenolic compounds, which makes anthocyanins hydrophilic and provides part of that nature to the polymer; on the other hand, the hydrophilic nature of both PVOH and anthocyanins contributes to increased permeability [47].

#### 3.5.3. Structural Properties

**Figure 5:**
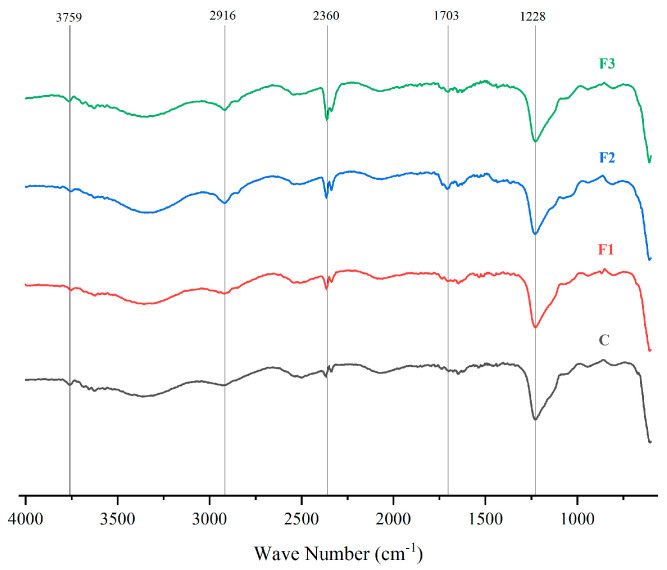
FTIR spectra of PVOH films with different concentrations of CAE (F1, F2, F3) and a control (C, pure PVOH).

Figure 5 presents the FTIR spectra of PVOH films with different CAE concentrations (F1, F2, F3) and a pure PVOH control (C). These spectra allow for the identification of molecular interactions between PVOH and CAE highlighting structural modifications in the films [48].

Changes in the characteristic bands were observed upon addition of CAE, indicating interactions between PVOH and CAE. The bands between 3758 cm^−1^ and 2916 cm^−1^ correspond to -OH and -CH stretching, respectively, showing intensity variations with CAE concentration [49,50], and show variations in intensity with increasing CAE concentration. In addition, peaks are observed at 1702 cm^−1^ and 1228 cm^−1^, indicate carbonyl (C=O) and ether (C-O) stretching, becoming more pronounced in F1, F2, and F3, confirming successful CAE incorporation into the PVOH matrix [51,52].

Similar studies present the following absorption peaks of the PVOH film: -OH stretching at 3272 cm^−1^; asymmetric and symmetric stretching of -CH_2_ groups at 2936 and 2902 cm^−1^ respectively; H-O-H bending of adsorbed water at 1644 cm^−1^; C=C stretching at 1538 cm^−1^; CH_2_ bending and waviness at 1415 cm^−1^ and 1324 cm^−1^, respectively [53].

A peak at 2360 cm^−1^ of the FTIR spectrum likely corresponds to atmospheric CO_2_ absorption during measurement, as it appears uniformly across all formulations [54,55].

The internal transmittance (Ti) and opacity values, presented in Table 6, confirm the high transparency of PVOH films, making them suitable for applications requiring light transmission.

Ti values increased as the anthocyanin extract content in the polymeric matrix increased, intensifying the red colour of the films and favouring light transmission, specifically at 450 nm. In addition, the films showed low opacity values, confirming their high transparency and ability to allow significant light to pass through. Thus, the films with higher internal transmittance (F2 and F3) are ideal for food packaging requiring high transparency such as fresh produce, while the control formulation might be preferred for those requiring higher light protection.

However, the films with CAE, especially F2, show higher transparency and lower opacity than the control film, indicating that the incorporation of CAE favourably influences the optical properties of PVOH films and could be ideal for packaging that requires product visibility while providing some light protection. On the other hand, F3, which has the highest amount of CAE, is the opaquest. Other studies have also attributed this increase in opacity to the presence of polyphenolic groups, which effectively absorb UV-Vis light [55]. Studies by Zhang et al. [56] on chitosan-anthocyanin-TiO_2_ films demonstrated that phenolic groups in anthocyanins enhance UV absorption, further supporting these findings.

**Figure 6:**
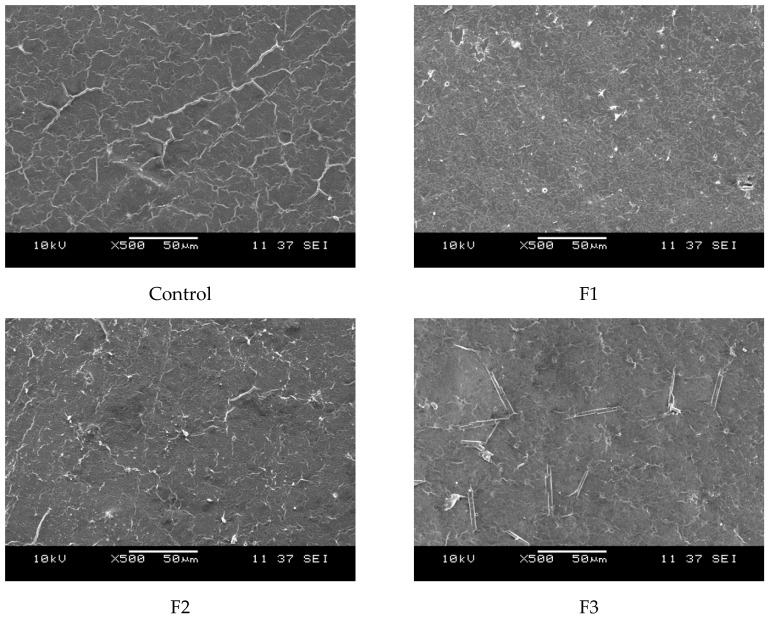
SEM micrographs of the cross-sections of the studied films at 500×: Control (0% CAE); F1 (10% CAE); F2 (20% CAE); and F3 (30% CAE).

On another hand, Figure 6 presents the surface morphology of the films as observed through scanning electron microscopy (SEM). The films exhibit a uniform polymeric matrix with minor deformations, likely induced by casting drying and cryofracture. Additionally, dispersed particles are visible within the continuous polymeric matrix, with their size increasing as the concentration of CAE in the films rises. These dispersed particles are likely from the plant extracts.

One key limitation in film preparation was PVOH’s low solubility in ethanol. Studies by Kim et al. [57] reported that PVOH has a low solubility in ethanol, quantified at 0.001 mg/mL, indicating that ethanol is not an efficient solvent for this polymer. Furthermore, Lee et al. (2023) [58] stated that in solutions containing more than 50% ethanol, PVOH solutions exhibited precipitation due to reduced solubility.

To address this issue, the films were filtered before drying, reducing undissolved PVOH residues. Previous studies, including those by Monne et al. and Zakaria et al. [59,60], confirm that PVOH’s semicrystalline structure and strong hydrogen bonding reduce its solubility in organic solvents, explaining these findings.

## 4. Conclusions

This study demonstrates that the concentration of anthocyanin extracts significantly influences their halochromic response, with lower concentrations exhibiting more rapid and pronounced colour changes. Corozo peel and pulp display distinct pH-dependent colour variations, confirming their potential as natural pH indicators.

The extraction process for corozo peel yielded a high anthocyanin content within a shorter extraction time, emphasising the importance of optimising the solid-to-solvent ratio, temperature, and extraction duration. However, the high sugar content in the pulp suggests the need for further process adjustments to improve extraction efficiency.

Analysis of PVOH films incorporating anthocyanin extract revealed that the extract concentration affects film colour, transparency, and surface morphology, while the moisture content and water vapor permeability remain largely unchanged. These results indicate that the films retain their optical and functional properties, supporting their application as pH-responsive materials.

Overall, this study highlights the potential of corozo extract as a sustainable source of anthocyanins for use in packaging and pH-sensitive materials. Future research should focus on optimising extraction processes and enhancing the functional properties of these films for commercial applications.

## Figures and Tables

**Table 1:** Central composite design factors for corozo pulp and peel extractions.

Experiment	Extraction Condition		
	t (min)	T (°C)	Solvent Amount (mL)
1	75	50	11.00
2	75	50	11.00
3	75	50	11.00
4	138	50	11.00
5	30	60	15.00
6	120	60	7.00
7	11	50	11.00
8	75	35	11.00
9	75	50	11.00
10	75	50	5.34
11	75	50	11.00
12	120	40	15.00
13	30	40	7.00
14	75	64	11.00
15	75	50	16.66

**Table 2:** Mass fractions of the studied formulations.

Formulation	PVOH	CAE	Glycerol
Control	0.833	0.000	0.167
F1 (10%)	0.161	0.806	0.032
F2 (20%)	0.089	0.893	0.018
F3 (30%)	0.062	0.926	0.012

**Table 3:** Extraction treatments and anthocyanin contents in corozo (*Bactris guineensis* L. H.E. Moore), predicted values vs. observed values in the laboratory.

Treatment	Extraction Condition	TAC Peel (mg EC_3_G/L)		
		Observed	Predicted	Relative Error
1	1:110, 75 min, 50 °C	17.92	19.81	0.10
2	1:110, 75 min, 50 °C	18.28	19.81	0.08
3	1:110, 75 min, 50 °C	18.17	19.81	0.08
4	1:110, 138 min, 50 °C	18.91	20.45	0.08
5	1:150, 30 min, 60 °C	34.61	35.55	0.03
6	1:070, 120 min, 60 °C	12.61	10.94	0.15
7	1:110, 11 min, 50 °C	17.70	19.17	0.08
8	1:110, 75 min, 35 °C	18.85	19.11	0.01
9	1:110, 75 min, 50 °C	22.04	19.81	0.11
10	1:5.343, 75 min, 50 °C	6.53	7.20	0.09
11	1:110, 75 min, 50 °C	20.58	19.81	0.04
12	1:150, 120 min, 40 °C	34.18	32.43	0.05
13	1:070, 30min, 40 °C	10.94	9.58	0.14
14	1:110, 75 min, 64 °C	19.91	20.52	0.03
15	1:16.66, 75 min, 50 °C	38.26	38.65	0.01
D	1:100, 30 min, 60° C	17.45	17.89	0.02

D: Treatment suggested by statistical optimisation with a desirability of 77.4%.

**Table 4:** CIELab scale colour values and gloss expressed in gloss units (GU) of the PVOH films.

Formulation	Brightness	Colour Parameters					
		L*	a*	b*	c*	h*	ΔE
Control	15.2 ± 4.02 ^a^	92.25 ± 0.16 ^a^	3.28 ± 0.15 ^d^	−0.6 ± 0.15 ^b^	3.35 ± 0.14 ^d^	1.78 ± 0.05 ^b^	--
F1	9.13 ± 3.39 ^b^	74.19 ± 8.16 ^b^	21.21 ± 8.51 ^c^	1.48 ± 1.38 ^b^	21.28 ± 8.59 ^c^	1.51 ± 0.03 ^a^	25.59 ± 11.71 ^c^
F2	15.26 ± 6.52 ^a^	63.10 ± 3.39 ^c^	36.92 ± 4.89 ^b^	0.69 ± 0.78 ^b^	36.93 ± 4.91 ^b^	1.55 ± 0.01 ^a^	44.54 ± 5.96 ^b^
F3	14.66 ± 7.15 ^a^	51.98 ± 10.95 ^d^	55.36 ± 13.33 ^a^	5.2 ± 4.46 ^a^	55.77 ± 13.71 ^a^	1.50 ± 0.08 ^a^	66.21 ± 17.63 ^a^

Values with different letters are significantly different (*p* < 0.05).

**Table 5:** Values of moisture content, water absorption capacity (WAC), water vapor permeability, and contact angle.

Treatment	MC (g Water/g Dry Film)	WAC (g Dry Film/g Absorbed Water)	WVP (g·mm/kPa·h·m^2^)	Contact Angle (°)
Control	0.24 ± 0.04 ^a^	0.39 ± 0.01 ^a^	0.56 ± 0.05 ^a^	26.33 ± 0.57 ^a^
F1	0.23 ± 0.0 ^a^	0.35 ± 0.01 ^ab^	0.57 ± 0.01 ^a^	24.00 ± 1.73 ^a^
F2	0.20 ± 0.01 ^a^	0.32 ± 0.03 ^b^	0.60 ± 0.04 ^a^	24.33 ± 1.52 ^a^
F3	0.22 ± 0.0 ^a^	0.32 ± 0.02 ^b^	0.56 ± 0.01 ^a^	24.66 ± 2.08 ^a^

Values with different letters are significantly different (*p* < 0.05).

**Table 6:** Structural properties, internal transmittance values at 450 nm and opacity (A_600_/mm) for PVOH and CAE films conditioned at 56%RH.

Treatment	Internal Transmittance (%)	Opacity (A_600_/mm)
Control	71.53 ± 4.89	2.73 ± 0.59
F1	75.35 ± 0.37	1.41 ± 0.02
F2	77.14 ± 0.01	1.10 ± 0.06
F3	78.44 ± 1.96	1.45 ± 0.13

## Data Availability

The original contributions presented in this study are included in the article. Further inquiries can be directed to the corresponding author.

## Abbreviations

The following abbreviations are used in this manuscript:

AS	Amount of solvent
AU	Absorbance unit(s)
CA	Contact angle
CAE	Corozo anthocyanin extract
CCD	Central composite design
CS	Solvent concentration
DF	Dilution factor
FCD	Face-centred cube design
FFS	Film-forming solution
FTIR	Fourier-transform infrared spectroscopy
GU	Gloss units
MC	Moisture content
MW	Molecular weight
PVOH	Polyvinyl alcohol
RH	Relative humidity
RSM	Response surface methodology
TAC	Total anthocyanin content
Ti	Inner transmittance
UV-Vis	Ultraviolet-visible spectroscopy
WAC	Water absorption capacity
WP	Water vapor permeability
WVP	Water vapor barrier properties
